# Counting vacancies and nitrogen-vacancy centers in detonation nanodiamond†
†Electronic supplementary information (ESI) available: (1) DND synthesis; (2) HRTEM and EELS characterization methods; (3) EELS simulation method; (4) supporting figures of EELS simulations; (5) soft-X-ray K-edge spectra of the DND; and (6) *ab initio* N-V center modeling method. See DOI: 10.1039/C6NR01888B



**DOI:** 10.1039/c6nr01888b

**Published:** 2016-04-28

**Authors:** Shery L. Y. Chang, Amanda S. Barnard, Christian Dwyer, Chris B. Boothroyd, Rosalie K. Hocking, Eiji Ōsawa, Rebecca J. Nicholls

**Affiliations:** a Leroy Eyring Center for Solid State Science , Arizona State University , Tempe , USA . Email: shery.chang@asu.edu; b Virtual Nanoscience Laboratory , CSIRO , Parkville , Australia; c Department of Physics , Arizona State University , Tempe , USA; d Ernst Ruska-Centre for Microscopy and Spectroscopy with Electrons , Forschungszentrum Jülich , Jülich , Germany; e College of Science Technology and Engineering , James Cook University , Townsville , Australia; f NanoCarbon Research Institute , Ueda , Japan; g Department of Materials , University of Oxford , UK . Email: rebecca.nicholls@materials.ox.ac.uk

## Abstract

Detonation nanodiamond particles (DND) contain highly-stable nitrogen-vacancy (N-V) centers, making it important for quantum-optical and biotechnology applications. However, due to the small particle size, the N-V concentrations are believed to be intrinsically very low, spawning efforts to understand the formation of N-V centers and vacancies, and increase their concentration. Here we show that vacancies in DND can be detected and quantified using simulation-aided electron energy loss spectroscopy. Despite the small particle size, we find that vacancies exist at concentrations of about 1 at%. Based on this experimental finding, we use *ab initio* calculations to predict that about one fifth of vacancies in DND form N-V centers. The ability to directly detect and quantify vacancies in DND, and predict the corresponding N-V formation probability, has a significant impact to those emerging technologies where higher concentrations and better dispersion of N-V centres are critically required.

## Abstract 2

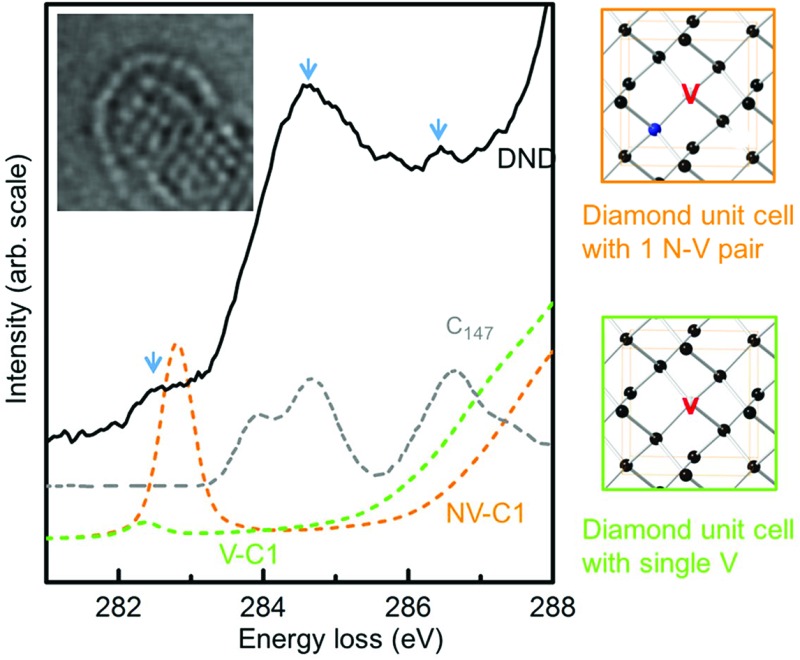
Vacancies and Nitrogen-vacancy centers in detonation nanodiamond particles can be detected and quantified.

Nitrogen-vacancy centers (N-V) in nanocrystalline diamond have been studied extensively for their interesting photoluminescence properties.^1,2^ Negatively-charged N-V centres (N-V^–1^) can emit visible light that is readily detectable, even at room temperature.^3^ Hence, nanodiamonds containing N-V centers have been used for quantum-optical applications and they are proven candidates for quantum information technologies based on solid-state qbits.^4,5^ In addition, the bio-compatibility of nanodiamond has seen emerging applications in biotechnology,^6–10^ such as spin imaging, and fluorescent biomarkers.

While there are a range of methods for producing nanodiamonds, detonation nanodiamond (DND), whereby high synthesis pressure is attained *via* a detonation process,^11^ is an economical method for producing nanodiamonds of small (typically <4 nm) and uniform size. On the other hand, while N-V centres in DND have been observed experimentally,^12,13^ their concentration is believed to be very low due to the small particle size. Efforts have been made to increase the N-V concentration *via* an increased vacancy concentration, *e.g.*, using high-energy irradiation.^14^ However, in the case of nanodiamond, it is far from clear whether this approach is effective, since the presence of planar defects and strain may influence the vacancy diffusion. Moreover, controlling and monitoring the production of vacancies is extremely challenging, and no technique currently exists for directly measuring the vacancy concentration in nanocrystalline materials.

Here we demonstrate that it is possible not only to detect, but to quantify, vacancies in DND using a combined experimental–theoretical approach. Then, using first-principles modelling, we show that the vacancy concentration is linked to the probability of forming N-V centers.

The measurement of vacancies is based on high-energy-resolution electron energy-loss spectroscopy (EELS) in a transmission electron microscope (TEM). Using EELS simulations, we show that vacancies and N-V centers in diamond can be identified by a well-defined peak in the pre-edge of the carbon K-edge spectrum. Atomic-resolution imaging is used to guide our interpretation of other pre-edge features. Then, the probability of forming N-V centres for a given particle size, and nitrogen and vacancy concentrations, is estimated using density functional tight-binding simulations and analytical calculations. Our work shows that N-V centers can exist in sub-4 nm DND under suitable nitrogen and vacancy concentrations.

Fig. 1(a) shows an atomic-resolution TEM image of a representative region of DND particles surrounded by vacuum. The image was obtained using a monochromated, spherical- (C_s_) and chromatic- (C_c_) aberration-corrected PICO TEM (FEI Co.) operated at an accelerating voltage of 80 kV. Here the C_c_ correction enables us to clearly resolve even the {311} spacing (1.07 Å) in the diamond cores. The low beam energy (80 keV) is below the knock-on threshold of diamond, enabling us to largely circumvent the adverse effects of beam damage. It is generally accepted that DND particles are composed of a diamond core surrounded by single or multiple fullerene-like shell(s). Fig. 1(b) shows such a particle. Its shell structure is interpretable as a surface relaxation effect, whereby the outmost (111) surface is displaced outwards non-uniformly to produce a curved surface, which is uncharacteristic of the reconstructed (111) surface of the bulk diamond. These observations are in agreement with both *ab initio* calculations of diamond nanoparticles^15,16^ and structures deduced from X-ray absorption spectroscopy.^15^ Fig. 1(c) shows that these relaxed surface structures persist in particles with multiple twins, a commonly observed defect in DND.

**Fig. 1:**
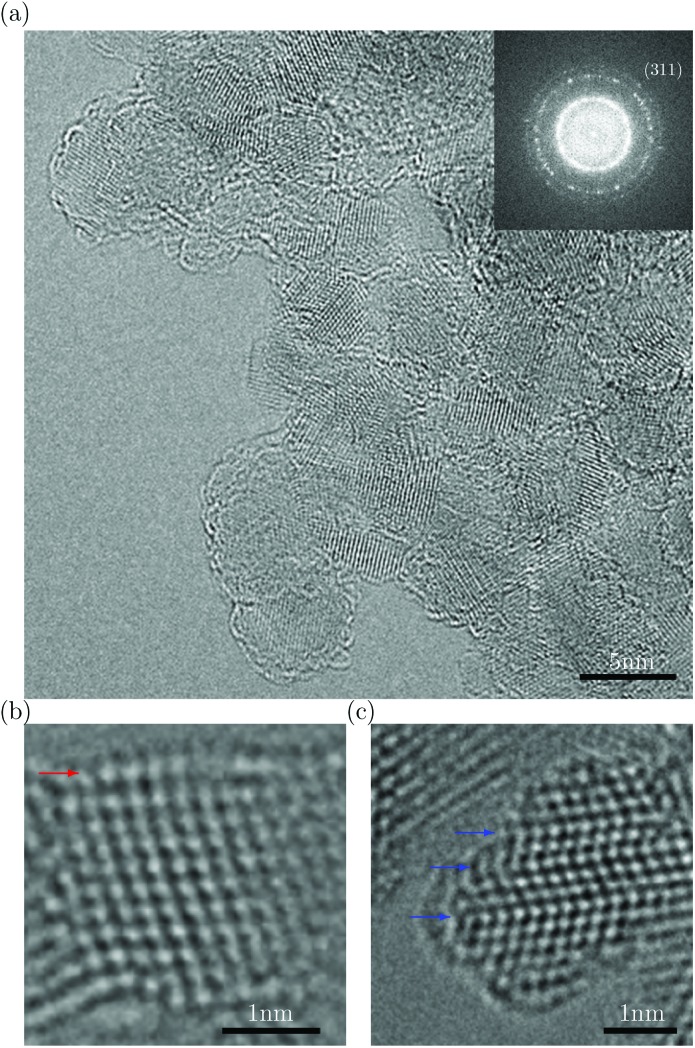
(a) C_s_–C_c_ corrected TEM image of a representative area of DND. The power spectrum of the image demonstrates 1 Å spatial resolution. (b) Detailed structure of a single DND particle with the relaxed and curved (111) surface indicated by the red arrow. (c) DND particle with multiple twins (indicated by blue arrows) with fullerene-like surfaces.

Although our HRTEM images reveal the atomic structures of surfaces and planar defects, the point defects within DND, *i.e.*, vacancies or N-V centers, cannot be directly visualised. To elucidate the possible presence of point defects, we use high-energy resolution EELS. Fig. 2(a) shows the EELS spectra of DND, together with reference spectra from bulk diamond, graphite and C_60_ fullerenes. Special care was taken to avoid exposing the surrounding amorphous carbon film on the TEM grid to the electron beam while taking the EELS spectra of the materials of interest. The spectrum of DND is very similar to that of diamond apart from the three additional pre-peaks. These pre-peaks are labelled A, B and C (282.5, 284.6 and 286.5 eV) in Fig. 2(b). Pre-peaks B and C are attributed to the fullerene-like surfaces of DND (shown in Fig. 1(b)), as evidenced by comparisons with both experimental spectra from the fullerene family^17,18^ and the simulated XAS spectra from the C_147_ particle^15^ (see Fig. 2(b)).

**Fig. 2:**
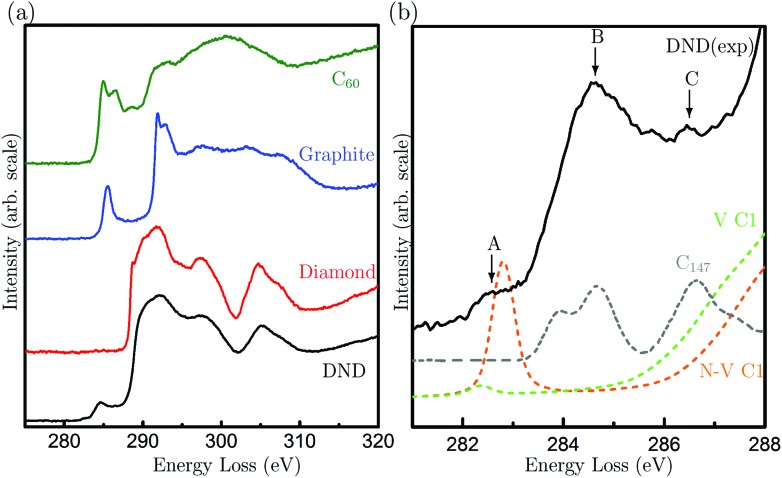
(a) C K-edge electron energy loss spectra of DND particles, bulk diamond powders, fullerene C_60_ and graphite; (b) the near edge structure of nanodiamond compared to the simulated spectra from a fully-relaxed C_147_ particle (reproduced from ref. 15) (C_147_, grey), a vacancy in the diamond lattice (V C1, green) and a nitrogen-vacancy pair in diamond (N-V C1, orange). The arrows indicate the three pre-peaks observed in DND.

To investigate the origin of the peak A, we have conducted EELS calculations based on density functional theory (DFT). While previous works^19–21^ have interpreted this peak as arising from surface dangling bonds of DND, here we find that such an interpretation cannot be supported: detailed calculations of spectra from diamond nanoparticles,^15^ and experimental spectra of reconstructed pristine diamond surface^22^ (all of which contain dangling bonds) do not contain this peak. Also, the surface layers of DND particles are fullerene-like, but experimental and simulated spectra from the fullerene family do not exhibit this peak. Line defects can be ruled out, since such defects are not observed in DND and they do not contribute to a pre-peak at this energy loss.^23^ We have also ruled out electron beam damage as the cause, since peak A is also present in our X-ray absorption spectra from the same sample (Fig. S1†). Hence, we focus our attention on point defects.

To this end, diamond models were constructed for an isolated vacancy (V) and an N-V pair, as shown in Fig. 3(a) and (b). Carbon K-edge spectra were calculated from first-principles using the plane-wave pseudopotential DFT code CASTEP^24^ in conjunction with the spectral code OptaDOS.^25^ All calculations were carried out using the GGA PBE functional and ultrasoft pseudopotentials. This method of EELS calculation, particularly on carbon systems, are accurate and have previously been shown to give excellent agreement between experimental and theoretical spectra.^18,26^ Fig. 3(c) shows the carbon K-edge spectra of perfect diamond and graphite, as well as spectra arising from the nearest neighbor atoms labeled C1 in the V and N-V models. Close attention has been paid to the energy alignment of these spectra, and also with the experimental spectra (Fig. S2†). We find that the spectra from N-V C1 and V C1 are very similar, except that the N-V C1 shows a pronounced peak at 282.8 eV, and V C1 shows a much smaller peak at a slightly lower energy of 282.4 eV.

**Fig. 3:**
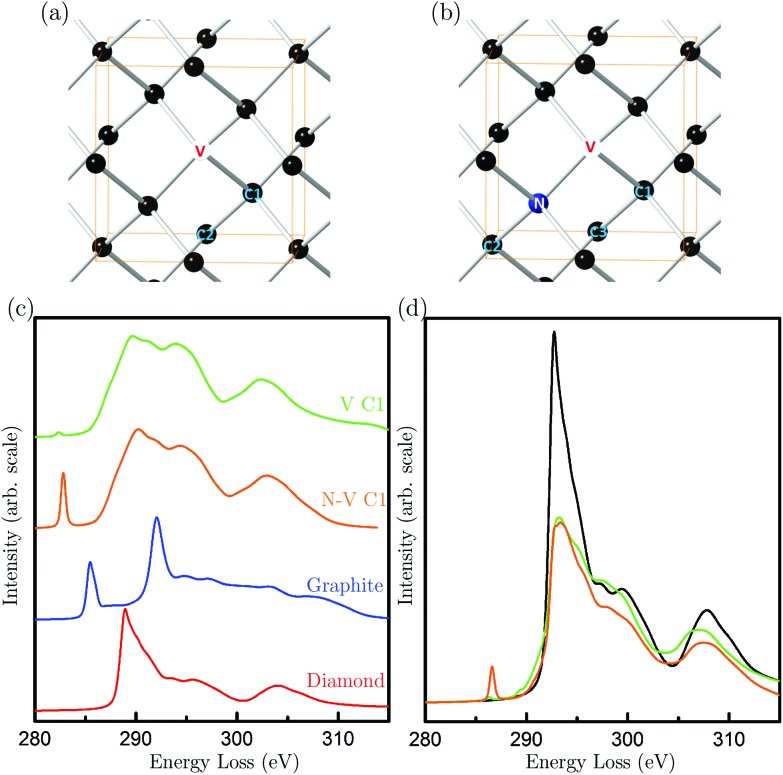
(a) Atomic model of an isolated vacancy (marked as V) in the diamond lattice; (b) atomic model of a nitrogen-vacancy pair in the diamond lattice. The unit cell of the pristine diamond is marked in (a) and (b) for reference; (c) simulated EELS spectra of a carbon atom of diamond (red), of graphite (blue) and the nearest neighbouring atom to the vacancy (V C1) as shown in (a), and the nearest neighbouring atom to the vacancy of the N-V pair (N-V C1) as shown in (b); (d) simulated EELS spectra of a diamond unit cell (black), a diamond unit cell with an isolated vacancy (green), and a diamond unit cell with a N-V pair (orange).

Spectra from the second- and third-neighbor atoms do not contain a pre-peak (Fig. S3†), and hence only those atoms nearest the vacancies give rise to such a peak. The peak positions are in excellent agreement with peak A in the experimental spectrum of DND (Fig. 2(b)). Our theoretical calculations suggest that the sensitivity to N-V centers is greater than that of isolated vacancies. The experimental energy resolution is, however, insufficient to differentiate between these two types of defects. Hence, our results strongly suggest that peak A arises from vacancy-containing point defects in DND, which could be either isolated vacancies or N-V pairs.

Having established that peak A arises from vacancies, we can use it to quantify the vacancy concentration in DND. This has been achieved by using the calculated spectrum of a diamond unit cell containing a vacancy (either N or N-V) as a reference spectrum (Fig. 3(d)). The vacancy concentration *f* is then given by the following formula (similar to that commonly used to quantify the degree of bonding hybridisation in carbon materials^27^):1
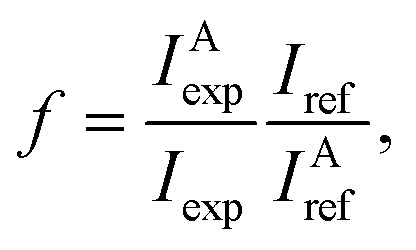



Hence the vacancy concentration is given by the ratio of the peak A intensity *I*Aexp and the carbon K-edge intensity *I*_exp_, normalised against that from the reference spectrum. Energy integration windows of 2 eV and 30 eV were used for peak A and the carbon K-edge, respectively, and multiple scattering effects were removed from the experimental spectrum. Using this approach, we estimate the vacancy concentration of DND to be in the range 0.4–1.3 at%, the lower and upper bounds corresponding to having all N-V pairs and all isolated vacancies, respectively.

If one considers DND as small particles of pristine diamond, then the estimated concentration of vacancies appears to be high because it could be argued that the vacancies are likely to diffuse to the surface. However, we observe that, despite the small particle size, DND contains a high concentration of planar faults (primarily twins), as well as dramatically-reconstructed surfaces. In previous work we have shown that the reconstructed surface of DND has a higher energy barrier that can trap vacancies.^28^ Therefore a higher concentration of vacancies is plausible.

Lastly, to establish the possibility that a fraction of the vacancies in DND belong to an N-V pair, we measured the N concentration using the N-K edge spectra, giving a concentration of 3 ± 0.2 at%. Then, using these parameters as input into a previously tested theoretical model,^28^ we can predict the probability of N-V pair formation for the measured range of vacancy concentrations. The calculations were carried out using the density-functional tight-binding method with self-consistent charges (SCC-DFTB). Fully relaxed nanodiamond particles of sizes corresponding to our experimental observations were used as initial configurations. The characteristic energies of defects in the core and shell regions where determined statistically, also accounting for the kinetic barriers to diffusion *ab initio*. The results are shown in Fig. 4. The calculation shown in Fig. 4 assume a particle size of 3.5 nm, however, the size dependence (not shown) is negligible. We also see that the uncertainty in the measured N concentration has little impact. These results show that approximately one fifth of the vacancies are likely to belong to an N-V pair. These results highlight the usefulness of ion implantation in generating N-V defects in nanodiamond, since the damage that is created during these activities is arguably more functional than the N that is implanted.^29^

**Fig. 4:**
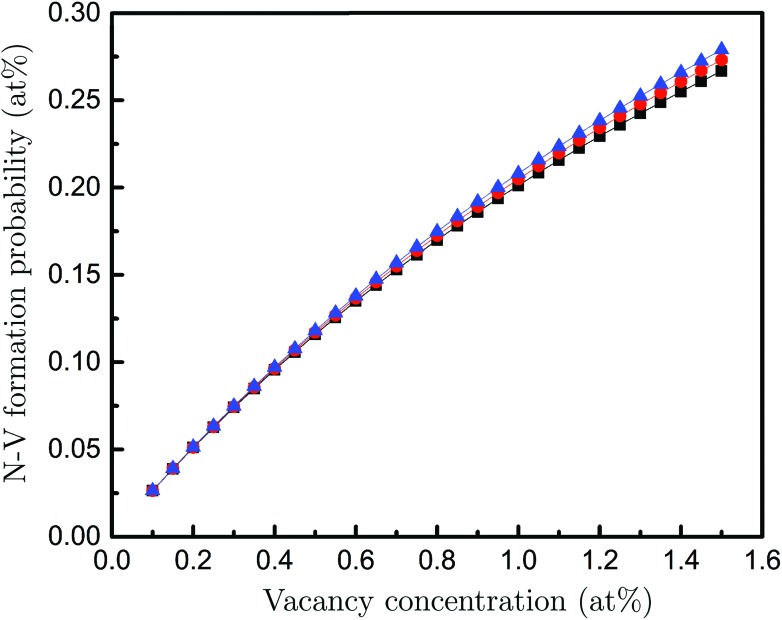
Probability of forming an N-V centre as a function of the vacancy concentration for the nitrogen concentrations of 2.8 (black), 3.0 (red) and 3.2 (blue) at%.

In summary, we have shown that vacancies in sub-4 nm DND can be measured quantitatively using simulation-aided EELS in a TEM. In addition, atomic-resolution TEM has revealed unambiguously the surface and planar-defect structures in DND, which in-turn guided our interpretation of pre-peak features in EELS. Based on the experimental results, our *ab initio* calculations predict that about 20% of the vacancies in DND form N-V centres. The ability to directly quantify the vacancy concentration in DND, and then predict the corresponding probability of N-V formation, should significantly aid the development of materials for biotechnology and quantum information technology applications, where higher concentrations and better dispersion of N-V centres are critically required.
