# Supplementary material for: Counting vacancies and nitrogen-vacancy centers in detonation nanodiamond
Source: Nanoscale. 2016 Apr 28;8(20):10548–52. doi: 10.1039/c6nr01888b (PMC5048336; doi:10.1039/c6nr01888b)
Supplement: Supplementary file 1 [file NR-008-C6NR01888B-s001.pdf]

Electronic Supplementary Material (ESI) for Nanoscale.
This journal is © The Royal Society of Chemistry 2016

Supporting Information

Counting vacancies and nitrogen-vacancy centers in detonation nanodiamond

Shery L.Y. Chang

Leroy Eyring Center for Solid State Science,

Arizona State University, Tempe, USA

Amanda S. Barnard

Virtual Nanoscience Laboratory, CSIRO, Parkville, Australia

Christian Dwyer

Department of Physics, Arizona State University, Tempe, USA

Chris B. Boothroyd

Ernst Ruska-Centre for Microscopy and Spectroscopy with Electrons,

Forschungszentrum Jülich, Jülich, Germany

Rosalie K. Hocking

College of Science Technology and Engineering,

James Cook University, Townsville, Australia

Eiji Ōsawa

NanoCarbon Research Institute, Ueda, Japan

Rebecca J. Nicholls

Department of Materials, University of Oxford, UK

I. DND SYNTHESIS

Detonation nanodiamond particles are provided by NanoCarbon Research Institute Co., Japan. The particles are prepared by a six-step procedure, including preliminary screening of the crude agglutinates, slurry preparation, beads-milling, intense supersonic treatment with sonotrod, centrifugal separation of uncrushable residue, and particle size distribution determination. Further details of each step can be found in Ref [1].

II. HRTEM IMAGING AND EELS USING MONOCHROMATED-CS/CC CORRECTED TEM

Monochromated, C_3-C_c corrected TEM, PICO, operated at 80 kV, was used to obtain high-resolution TEM image and EELS spectra of DND. For the imaging, spherical aberration C_3 was corrected to -1 μm and C_c to 2 μm, giving a theoretical resolution of 0.8 Å resolution. This is significantly lower compared to interments without C_c correction, typically gives about 1.8 Å resolution for 80kV.

Monochromator was excited to give 0.2 eV (FWHM) energy resolution for EELS. Absolute energy level and the energy dispersion were both calibrated in order to align the spectra for DND and other reference materials.

III. SIMULATION OF EELS

A. Details of DFT calculations

EELS simulations were carried out from first principles using a plane-wave pseudopotential density functional theory (DFT) code (CASTEP [2]) together with the OptaDOS [3] spectral simulation code. Unit cells were constructuted for pure diamond and graphite. For the N-V and diamond-V models, geometry optimisations were based on 3x3x3 supercells of face-centered cubic diamond structures. All CASTEP calculations were carried out with the GGA PBE functional and ultrasoft pseudopotentials. A cut-off energy of 520 eV and k-point spacing of 0.05 Å^{-1} were used for the geometry optimisations and the models were optimised until the forces on each atom were less than 1×10^{-3} eV Å^{-1}.

A cut-off energy of 520 eV and k-point spacing of 0.01 $\AA^{-1}$ were used for the EELS

simulations and the distance between core-holes was at least 10.7 Å for the diamond-based models. Increasing these parameters for diamond was found to have no visable effect on the simulated spectra. For graphite, the spacing of the core-holes within the graphitic plane was 12.3 Å which has previously been found to be far enough apart for the core-hole interactions to be negligable in graphene [4]. The spacing of the core-hole out of the graphitic plane was 6.7 Å, an increase in this parameter was found to have a negligible effect on the simulated spectra. In order to compare the simulated data with experiment, broadening due to instrumentation and life-time effects was included in the simulation. The theoretical spectrum was convolved with a Gaussian of 0.4 eV (full width at half the maxium, FWHM) to represent the instrumentation broadening and a Lorenzian with FWHM of $(0.2 + E/10)$, where E is the energy above the Fermi level, to represent the life-time effects.

B. Alignment of experimental and theoretical spectra

The simulated spectra for the different structures have been put on the energy loss scale using the method outlined by Mizoguchi et al. [5]. Although this method is good for aligning spectra from carbon atoms in different environments, the energy of the simulated spectra is not exactly the same as that of the experimental data. By shifting the simulated data by -3.8 eV, it is possible to obtain a good match with the experimental data for both diamond and graphite, as shown in Fig. S1 and so this shift has been applied to all simulated spectra.

IV. EELS SIMULATION FOR OTHER CARBON ATOMS SURROUNDING VACANCY

EELS simulation for the carbon atoms, C1 and C2 in the vacancy model shown in Fig. 3(a) and atoms C1, and C2 in the N-V model shown in Fig. 3(b). It can be seen that only the nearest carbon atoms C1 in both models show a pre-peak at the energy loss near the experimental pre-peak A. The other carbon atoms surrounding the vacancy exhibit features strongly resembling diamond, suggesting that those atoms do not experience bonding changes due to the presence of vacancy.

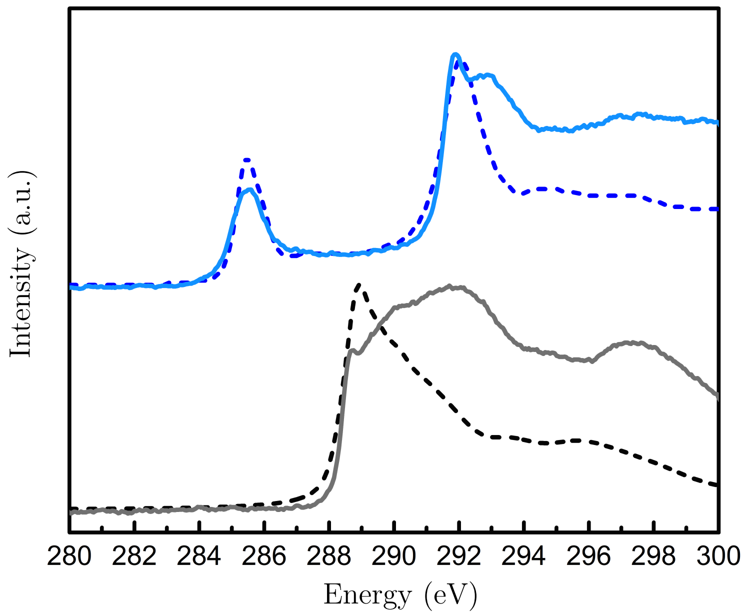

FIG. S1. Simulated (dotted lines) carbon K-edges for diamond and graphite aligned with experimental data (solid lines). The black lines represent diamond and blue lines graphite.

V. CARBON K-EDGE X-RAY ABSORPTION SPECTROSCOPY SPECTRA OF DND

The possible electron beam induced structural changes was investigated using soft x-ray XAS of C K edge.
Soft x-ray beam line in Australia Synchrotron has been used to acquire carbon K-edge spectra of DND. Fig. S3 shows the near edge structure of C K edge from the same sample used for TEM investigations.

VI. PREDICTION OF PROBABILITIES OF N-V CENTRE USING DFTB CALCULATIONS

The probability of observing an N-V pair in DND (bulky diamond) was calculated based on the method detailed in Ref [9]. In this work, fully relaxed clean and hydrogen passivated nanodiamond particles of the size 3.5 nm were used as initial configurations, and the various point defects were substituted for carbon atoms located along specific substitution paths within the lattice. The paths extend from the centro-symmetric atom to different

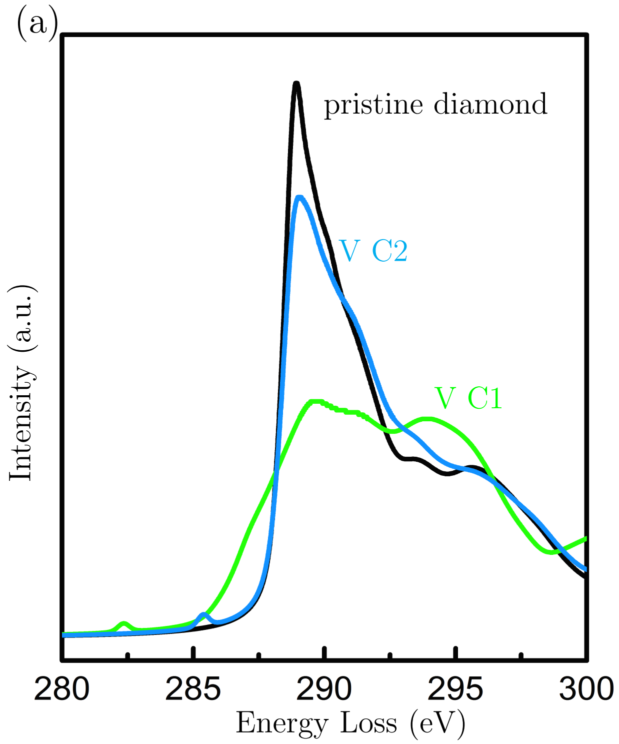

FIG. S2. Simulated EELS spectra of non-equivalent carbon atoms surrounding the vacancy for (a) an isolated vacancy in diamond and (b) a N-V pair in diamond. The models and the carbon atoms are labelled as shown in Fig. 3.

points on the surfaces, edges and corners. Since part of the study requires >150 individual simulations on entire (experimentally realistic) nanodiamond structures, we have used the density functional tight-binding method with self-consistent charges (SCC-DFTB), which was implemented in the DFTB+ code [6], to perform the individual calculations [7, 8]. The SCC-DFTB is an approximate quantum chemical approach where the Kohn–Sham density functional is expanded to second order around a reference electron density. The reference density is obtained from self-consistent density functional calculations of weakly confined neutral atoms within the generalized gradient approximation (GGA). The confinement potential is optimized to anticipate the charge density and effective potential in molecules and solids. A minimal valence basis set is used to account explicitly for the two-center tight-binding matrix elements within the DFT level. The double counting terms in the Coulomb and exchange–correlation potential, as well as the intra-nuclear repulsion are replaced by a universal short-range repulsive potential. All structures have been fully relaxed with a

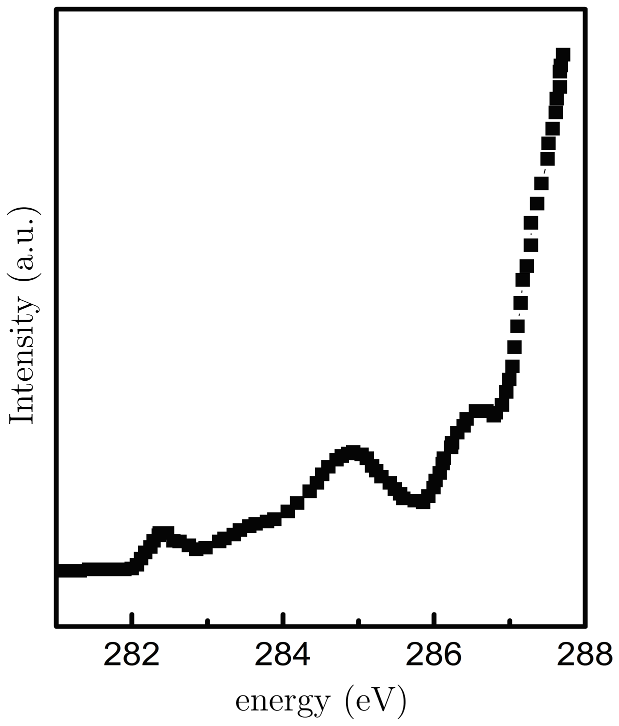

FIG. S3. C K-edge X-ray absorption spectroscopy spectra of the sub-4 nm DND.

conjugate gradient methodology until forces on each atom was minimized to be less than 10^{-4} a.u. (i.e. $\approx$5 meV/Å). In all the calculations, the "PBC" set of parameters is used to describe the contributions from diatomic interactions of carbon. The probability of observing an N-V pair in DND (bulky diamond) was calculated based on the formula described in Ref [9], where the characteristic energy of defect E_d in the sp^3-bonded core region and that in the sp^2-bonded shell region where determined statistically, and with the kinetic barriers to diffusions were calculated *ab initio*.

---

[1] L. Y. Chang, E. Ōsawa, and A. S. Barnard, Nanoscale **3**, 958 (2011).

[2] S. Clark, M. Segall, C. Pickard, P. Hasnip, M. Probert, K. Refson, and M. Payne, Z. Kristallogr. **220**, 567 (2005).

[3] A. J. Morris, R. J. Nicholls, C. J. Pickard, and J. R. Yates, Comp. Phys. Comm. **185**, 1477 (2014).

[4] R. J. Nicholls, A. T. Murdock, J. Tsang, J. Britton, T. J. Pennycook, A. Koos, P. D. Nellist, N. Grobert, and J. R. Yates, ACS Nano **7**, 7145 (2013).

[5] T. Mizoguchi, in *Scanning transmission electron microscopy of nanomaterials*, edited by N. Tanaka (Imperial College Press, London, 2014).

[6] B. Aradi, B. Hourahine, and T. Frauenheim, J. Phys. Chem. A **111**, 5678 (2007).

[7] D. Porezag, T. Frauenheim, T. Köhler, G. Seifert, and R. Kaschner, Phys. Rev. B **51**, 12947 (1995).

[8] T. Frauenheim, G. Seifert, M. Elstner, T. Niehaus, C. Köhler, M. Amkreutz, M. Sternberg, Z. Hajnal, A. Di Carlo, and S. Suhai, J. Phys.: Condens. Matter **14**, 3015 (2002).

[9] A. S. Barnard, Mater. Horizons **1**, 289 (2014).
